# Impairment of Auditory-Motor Timing and Compensatory Reorganization after Ventral Premotor Cortex Stimulation

**DOI:** 10.1371/journal.pone.0021421

**Published:** 2011-06-29

**Authors:** Katja Kornysheva, Ricarda I. Schubotz

**Affiliations:** 1 Motor Control Group, Institute of Cognitive Neuroscience, University College London, London, United Kingdom; 2 Motor Cognition Group, Max Planck Institute for Neurological Research, Cologne, Germany; 3 Institute of Psychology, University of Münster, Münster, Germany; Royal Holloway, University of London, United Kingdom

## Abstract

Integrating auditory and motor information often requires precise timing as in speech and music. In humans, the position of the ventral premotor cortex (PMv) in the dorsal auditory stream renders this area a node for auditory-motor integration. Yet, it remains unknown whether the PMv is critical for auditory-motor timing and which activity increases help to preserve task performance following its disruption. 16 healthy volunteers participated in two sessions with fMRI measured at baseline and following rTMS (rTMS) of either the left PMv or a control region. Subjects synchronized left or right finger tapping to sub-second beat rates of auditory rhythms in the experimental task, and produced self-paced tapping during spectrally matched auditory stimuli in the control task. Left PMv rTMS impaired auditory-motor synchronization accuracy in the first sub-block following stimulation (p<0.01, Bonferroni corrected), but spared motor timing and attention to task. Task-related activity increased in the homologue right PMv, but did not predict the behavioral effect of rTMS. In contrast, anterior midline cerebellum revealed most pronounced activity increase in less impaired subjects. The present findings suggest a critical role of the left PMv in feed-forward computations enabling accurate auditory-motor timing, which can be compensated by activity modulations in the cerebellum, but not in the homologue region contralateral to stimulation.

## Introduction

An important research goal in basic and clinical neuroscience is to understand recovery of cognitive function. While some progress has been made in uncovering mechanisms of functional reorganization of motor, visuo-motor and partly speech recovery, compensatory mechanisms during auditory-motor integration remain largely unknown. Yet, the ability to accurately time movements on the basis of auditory input is essential in a variety of domains and situations, such as speech, singing and synchronizing to music, but also in environments with missing or only scarce visual information.

As a part of the dorsal auditory stream, the ventral premotor/frontal opercular region (PMv) has been shown to be a node for auditory-motor integration, specifically with regard to sequential auditory patterns like speech or music [1]–[3], in which timing is essential. Both its anatomical position and its functional properties suggest that this region may play a critical role in auditory-motor timing.

The inferior portion of the PMv and the adjacent frontal operculum are reciprocally connected to auditory areas in the superior temporal gyrus via the arcuate fasciculus (AF) [4], [5]. Humans are known to have an enhanced AF compared to other primate species [6] - a finding that strongly corresponds to the notion that humans are the only primate species exhibiting vocal learning and speech [7]. In primates, the PMv has been shown to possess direct corticospinal outputs and project to the primary motor cortex via association fibres [8], [9], as well as to its homologue, the contralateral PMv [10].

The PMv has been proposed to provide a common platform for timing, both perceived and produced [11]. The former is substantiated by neuroimaging studies involving sub-second temporal estimation [12], [13] and rhythmic sequence prediction tasks [14]–[18]; the latter by auditory-motor synchronization [19]–[22] and vocal imagery tasks [17], [23], [24]. Accordingly, the PMv has been suggested to be a part of a network that enables sensorimotor feed-forward prediction of both self-generated (re-afferent), as well as externally-generated (ex-afferent) events in the sub-second range [11]. However, other areas such as the PMd and the cerebellum have been associated with paced motor timing [25]–[29], as well, and the critical contribution of the PMv in auditory-motor timing is still under discussion [2].

In addition to the role of PMv in auditory-motor timing, it remains unclear, which degenerate set of brain areas may help to preserve auditory-motor timing performance following its disruption. Evidence from stroke and repetitive transcranial magnetic stimulation (rTMS) suggests that interhemispheric compensation may play an important role in motor, visuo-motor and speech recovery. However, this evidence is contradictory. Some studies report compensatory plasticity in the non-dominant hemisphere, when it comes to motor [30], visuo-motor [31] and speech functions [32]. Others, however, demonstrate that the contralesional hemisphere does not support behavioral compensation, and can even be maladaptive [33]–[35].

In the current study, rTMS was combined with subsequent fMRI to examine the critical role of the left PMv in auditory-motor timing and investigate mechanisms of compensatory short-term functional reorganization that can reduce a negative behavioral effect of PMv interference. We hypothesized that rTMS over the left PMv (i) disrupts the accuracy of auditory-motor timing, (ii) triggers task-specific activity increase in the homologous right PMv, and that (iii) the latter effect is compensatory – the higher the activity increase, the smaller the effect of left PMv stimulation across subjects.

Subjects had to synchronize left and right index finger tapping to the variable beat rate of auditory rhythms following rTMS over PMv as opposed to no rTMS. In accordance with Repp [36], variation of tap-to-beat asynchrony, was taken as a measure of auditory-motor timing accuracy. To ensure that the behavioral change is not just an unspecific effect of rTMS, subjects also participated in a control session with rTMS over the left angular gyrus/parieto-occipital lobe (AG). To probe the functional specificity of rTMS, we assessed motor timing variability in a control task, in which subjects produced self-paced tapping to spectrally matched auditory stimuli, and evaluated measures related to motor output, overall auditory-motor coupling and attention to the task.

## Methods

### Ethics statement

All subjects gave informed written consent to participate in this study. Experiments were approved by the Ethics Committee of the Medical Faculty, University of Cologne, Germany.

### Subjects

Sixteen healthy volunteers (mean age 24.8, range 22–29 years, eight females) participated in the study. All subjects were right-handed according to the Edinburgh Inventory of Manual Preference [37]. None of them were professional musicians. Their rhythm perception ability ranged from 23 to 30 (mean: 26.9; SE: 0.55) on a scale of 30 (online version of the rhythm test from the Montreal Battery of Evaluation of Amusia (MBEA), http://www.delosis.com/listening/home.html). Therefore, all subjects were within two standard deviations of the population mean (Peretz et al. [38]; cf. MBEA norms update 2008, http://www.brams.umontreal.ca/plab/publications/article/57). All subjects were naïve concerning the hypothesis of this study. However, 6 of 16 participants encountered the stimulus material for the second time, previously participating in a perceptual rhythm judgment experiment [39] or perceptual rhythm judgment pilot. None of the subjects had any history of medical or psychiatric disease or contraindication to TMS [40], [41].

### Stimuli and Tasks

**Figure 1:**
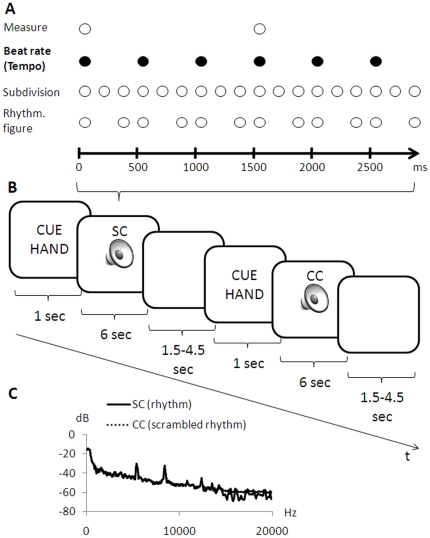
Stimulus material and trial structure. **A**: The auditory stimulus was determined by the factors beat rate (tempo/inter-onset-interval of beats; 1.7, 2.0 or 2.5 Hz), measure (the grouping of beats), beat subdivision (elements per beat), rhythmic figure and a factor unrelated to sub-second timing, timbre (spectro-temporal configuration of the sound stimulus), that varied on two or three levels respectively (cf. stimulus examples: Sounds S1, S2, S3, S4, S5, S6). Beat rate (filled circles) served as a cue for auditory-motor synchronization. The depicted rhythm example possesses a middle tempo with three beats per measure, three elements per beat and a repetitive rhythmic figure containing a long, followed by a short interval. **B**: Each trial started with an auditory cue (sinusoidal tone of either 400 or 1200 Hz, assignment counterbalanced across subjects), indicating whether to tap with the right or the left index finger, followed by the stimulus and a pause, which varied depending on the jitter times. In the synchronization condition (SC) subjects were instructed to tap to the respective periodic beat of the musical rhythm with the right or the left index finger according to the preceding cue. In CC subjects were presented with scrambled versions of musical rhythms (randomized 10 ms segments of the respective rhythms). The subjects' task was to tap regularly in a self-paced manner. **C**: The spectrum of the scrambled rhythms in CC closely matched that of the rhythms in the SC (cf. trial examples: Sound S7).

In the experimental condition, participants were presented with auditory musical rhythms consisting of drum sounds that were generated with the Microsoft Software Wavetable Synthesizer (GM drum map). The rhythmical stimuli have been previously used in a functional MRI and a TMS study [39], [42]. Each auditory rhythm had five properties – beat rate (slow - 1.7 Hz/100 beats per minute, (BPM), middle - 2.0 Hz/120 BPM and fast - 2.5 Hz/150 BPM), measure (beat grouping; 3, 4, 5 beats; 3/4, 4/4, 5/4 meter in musical notation), beat subdivision (3, 4, 5 elements per beat; eighths note triplet, four sixteenth notes and sixteenth note quintuplet in musical notation), rhythmic figure (long interval – short interval, short interval – long interval; dotted note and syncopation in musical notation), and timbre (“wooden” – predominantly wooden drum instruments), “metallic” – (predominantly metallic drum instruments); two versions of each timbre) – that varied orthogonally on two or three levels, respectively (cf. Fig. 1A; examples: Sounds S1, S2, S3, S4, S5, S6). Beat rate varied within the range of the preferred “tempo-octave” of contemporary dance music (Moelants 2003; van Noorden and Moelants 1999). In combination with the other four counterbalanced properties of the musical rhythms (beat subdivision, beat grouping, rhythmic figure, timbre), there was a pool of 216 possible permutations. Each rhythm was encountered only once - only 54 of the 216 possible permutations were presented in each of the four scans (cf. trial description below). Note that the factors beat grouping, beat subdivision, rhythmic figure and timbre were counterbalanced across beat rates, thus having no systematic effect on auditory-motor synchronization accuracy measured across beat rates. An important advantage of these types of stimuli is that in comparison to most studies that use isochronous metronome clicks for auditory-motor synchronization tasks, the current stimuli more closely resemble musical rhythms, and thus can be regarded as more ecologically valid cues for auditory-motor synchronization, while at the same time being experimentally controlled.

Subjects had two tasks: a synchronization condition (SC) and a control condition (CC). In the SC they were instructed to tap to the respective periodic beat of the musical rhythm with the right or the left index finger according to the preceding cue (sinusoidal tone of either 400 or 1200 Hz, assignment counterbalanced across subjects). In the CC they were presented with scrambled versions of musical rhythms (randomized 10 ms segments of the respective rhythms), which spectrally closely matched the stimuli in the SC (Fig. 1C), but lacked beat or any other type of a regular temporal structure. The subjects' task was to tap regularly in a self-paced manner, with the right or the left index finger according to the cue at the beginning of the trial. On the behavioral level, the CC served to evaluate motor timing, as opposed to auditory-motor timing. On the fMRI level, the CC served to subtract out spectral acoustic input, as well as motor output, hence allowing to isolate activation related to auditory-motor timing by comparison between SC and CC. Cues for left and right finger tapping were equally distributed across SC and CC trials. Subjects tapped on the respective index finger button of a fMRI-compatible bimanual serial response box (Current Design). The remaining buttons on the response box were covered by a custom-made plastic shield.

Each trial (10 s) started with an auditory cue (1 s), indicating whether to tap with the right or the left index finger, followed by the stimulus (6 s) and a pause, the length of which was variable (0.5–3.5 s) depending on the jitter times (0, 500, 1000, or 1500 ms) (Fig. 1B).

Since an inhibitory effect of rTMS usually does not outlast 20–30 minutes after the end of stimulation [43], the experiment lasted 20 minutes, during which 120 trials were presented in a pseudorandom fashion: 54 in the SC and the CC condition, respectively, as well as 12 in the resting condition (RC). To capture a possible recovery of synchronization accuracy after rTMS during this time range [31], [44] all conditions and levels of tempo and timbre were equally distributed across each of the four sub-blocks of 5 minutes, respectively. We used 4 different trial randomizations matching the above criteria.

### Procedure

All sixteen subjects participated in two rTMS-fMRI sessions with rTMS over either the left PMv or the left AG (rTMS control), respectively. The PMv and AG sessions were carried out at one-week intervals and their order was counterbalanced across participants. Each subject practiced the task directly prior to the first rTMS-fMRI session and practice was refreshed briefly prior to the second session. Each session started with a training containing example trials (9 trials SC and 9 trials CC), which were randomly chosen from the pool of stimuli for each subject and counterbalanced for tempo. This training had the purpose to familiarize the subjects with the task and the musical rhythms, as well as the range of tempos.

**Figure 2:**
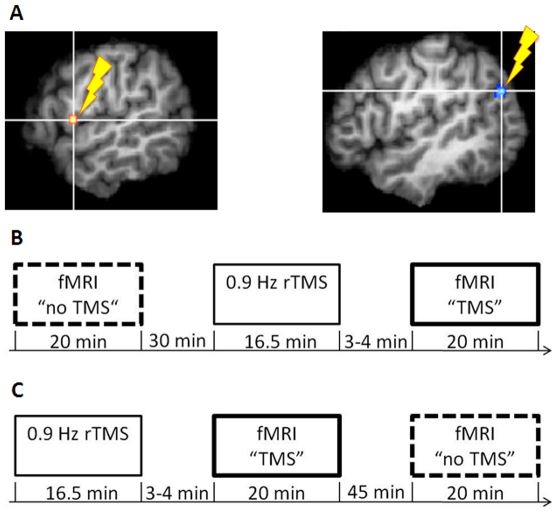
Stimulation sites and session procedure. **A:** Each subject participated in two rTMS-fMRI sessions separated by one week, in which rTMS was performed either over the left PMv or the left AG (rTMS control), respectively. The stimulation sites were chosen on the basis of Kornysheva et al. [59]. **B:** In one half of the subjects, each session started with an fMRI scan not preceded by rTMS (“fMRI no TMS” first), **C:** in the other half the fMRI scan following rTMS came first (“fMRI TMS” first)). In the latter case, the second fMRI scan (“no TMS”) was performed following a 45 minutes interval, during which the subjects stayed in a room adjacent to the MRI scanner room.

During each session subjects underwent two fMRI scans, one of which was preceded by 0.9 Hz rTMS over either the left PMv or the left AG. To exclude a learning effect, the scan order was counterbalanced across rTMS-sites and participants: In half of the subjects and sessions, respectively, the fMRI scan following rTMS came first (Fig. 2C). In this case, the second fMRI scan was performed following a 45 minutes interval, during which the subjects stayed in a room adjacent to the MRI scanner room. The four scans will are referred to as follows: “PMv TMS” (scan directly preceded by rTMS over PMv), “PMv no TMS” (scan not directly preceded by rTMS over PMv), “AG TMS” (scan directly preceded by rTMS over AG), “AG no TMS” (scan not directly preceded by rTMS over AG).

The fMRI scan following rTMS started 3:34 min (SE 0:07) after the end of PMv stimulation and 3:16 min (SE 0:03) after the end of AG stimulation. The 18 s difference between the PMv and AG sessions was significant (t = 2.6; *p*<.05, paired t-test). It most probably occurred due to the location of the stool that supported the experimenter during the administration of TMS pulses with respect to the room exit and the position of the wheelchair. To the best of our knowledge, there were no systematic differences with how the subjects were handled and how the experimenter responded to the subjects. Importantly, since fMRI after PMv stimulation started later than after AG, a possible effect of PMv stimulation on behavior or BOLD signal cannot be explained by a temporal proximity and stronger influence of rTMS. During the interval between the end of rTMS and the beginning of the fMRI scan, subjects were asked to interact with the experimenters as little as possible. They were moved with a wheelchair to the adjacent MRI scanner room and were only required to get onto the scanner bed.

### Site Localization

Stimulation targets (Fig. 2A) were chosen on the basis of a preceding fMRI study involving the same auditory rhythms [42] conducted with a different group of subjects. The PMv site was defined by the peak voxel in the left ventral premotor cortex (PMv) activated during auditory rhythms with preferred beat rate, as well as during a beat rate (tempo) judgment task (Talairach coordinate: −50 4 12). The control site (AG) was defined by the peak voxel activated in the left inferior parietal cortex for rest against all conditions involving musical rhythms (Talairach coordinate: −44 −68 30). The distance of the TMS coil to the left ear was approximately the same for the two target sites, ensuring a comparable amount of exposure to the TMS noise prior to the experiment. None of the subjects reported a difference between the sessions with regard to TMS noise intensity.

An individual high resolution T1-image (3D MDEFT, data matrix: 256×256×128) was acquired for each subject during a preceding scanning session. This 3D data set was transformed to Talairach stereotactic space [45]. The respective contrast images from the preceding fMRI study were overlaid on each transformed individual 3D data set. The peak voxels were marked by crosshairs on the axial, coronal and sagittal planes, respectively. Subsequently, the stimulation targets were set manually on the T1-image according to the individual anatomical landmarks surrounding the crosshairs on the transformed 3D data set.

### TMS stimulation

Stereotaxic frameless neuronavigation was obtained by the eXimia NBS system Version 2.1.1 (Nexstim, Helsinki, Finland). Coil tilting was tangential to the skull and current direction was perpendicular to the central sulcus. Online neuronavigation was used to maintain the targeted tilting and direction of the TMS coil across stimulation.

TMS was applied with a biphasic Nexstim Eximia TMS with a figure-of-eight-coil (diameter: 50 mm). Motor threshold was determined at each session prior to rTMS in the right first dorsal interosseus muscle. Electromyographic (EMG) signals were recorded by surface electrodes placed in a belly-tendon montage over the target muscle. The EMG signal was amplified, filtered with a 0.5 Hz high pass filter and digitized using a PowerLab 26 T Myograph and the “Scope” software package Version 3 (ADInstruments Ltd, Dunedin, New Zealand). The resting motor threshold (RMT) was assessed by means of the maximum likelihood method as suggested by Awiszus (2003; TMS Motor Threshold Assessment Tool (MTAT) 2.0, Awiszus F & Borckardt JJ, Brain Stimulation Laboratory, Medical University of South Carolina, USA, http://www.clinicalresearcher.org/software.htm), which has been suggested to be more accurate with the same number of stimuli [46]–[48] in comparison to techniques proposed by Rossini et al. [49], Rothwell et al. [50] or Mills and Nithi [51]. Peak-to-peak amplitudes exceeding 50 µV were regarded as motor evoked potentials.

Stimulation intensity was 90% of the individual resting motor threshold (RMT), with a mean stimulation intensity of 33.6% (1.6% SE) of maximum stimulator output in the PMv session and 35.1% (2.1% SE) in the AG session, the difference between the sessions being not significant (t = −1.5; *p* = .19, paired-samples t-test). There was a significant correlation between the RMT in the two sessions (*r* = 0.89; *p*<.001). For each of the sites stimulated, 900 pulses were applied at a frequency of 0.9 Hz (train duration 16.5 min). A stimulation frequency slightly below the standard 1 Hz stimulation was chosen to exclude potential interference of rTMS noise with the 2 Hz beat rate of 1/3 of the musical rhythms in the subsequent experiment (cf. Stimuli and Tasks).

### Behavioral analysis

#### Auditory-motor timing variability: (CV_SC_)

In the synchronization condition (SC) the subjects were instructed to synchronize their taps to the beat of the presented auditory rhythms. To assess the effect of TMS on auditory-motor timing variability, the coefficient of variation (CV_SC_) was computed with regard to the tap-to-beat asynchrony across SC trials. CV_SC_ of absolute tap-to-beat asynchrony A across SC trials was defined as follows:
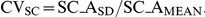
To make asynchrony comparable across rhythms with different beat rates, the absolute asynchrony A_i_ of each tap onset T_i_ minus the beat onset B_i_ was calculated as percent of inter-onset-interval (IOI) of consecutive beats:
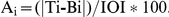
Average asynchrony A in percent IOI was calculated for each SC trial. Subsequently, CV_SC_ was determined across trials of each scan, as well as each sub-block of five minutes as outlined above.

Taps within the boundary of ±40% of the respective IOI around the beat onset were considered. This relatively wide criterion was chosen to account for the hypothesized increase in variability of asynchrony after rTMS over the left PMv. Only tap asynchronies relative to the third and following beats in each trial were taken into consideration, since a minimum of two consecutive beats is necessary to extrapolate the beat rate of an isochronous cue.

Since effects of rTMS stimulation can be expected to be most pronounced at the beginning of the measurement following stimulation and to cease towards the end (O'Shea et al., 2007), time-dependent effects within each scan were considered. A repeated measures ANOVA with the factors SITE (PMv/AG), TMS (no TMS/TMS) and TIME (1^st^/4^th^ sub-block) was performed. In case significant interactions between SITE, TMS and TIME were present (p<0.05), post-hoc Bonferroni corrected t-tests were computed for CV_SC_ to test for differences between TMS and no TMS sub-blocks in PMv and AG sessions, respectively.

#### Motor timing variability: CV_CC_

In the control condition (CC) the subjects were instructed to produce regular self-paced tapping to scrambled rhythms. To assess the effect of TMS on motor timing variability, the coefficient of variation (CV_CC_) was computed by taking the self-paced tapping frequency of the respective trial into account. The tap-to-tap' asynchrony was computed across CC trials where tap' T_ii_ is the expected time at which a tap T_i_ should occur according to the mean inter-tap-interval ITI_mean_ during the respective scrambled rhythm.

The mean inter-tap-interval ITI_mean_ was calculated as follows:
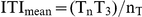
where n_T_ is the number of taps occurring in the time between the third tap T_3_ and the last tap T_n_ during the presentation of the scrambled rhythm.

The onset T_ii_ of each expected tap occurring between the third tap T_3_ and the last tap T_n_ were calculated as
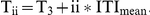
V_CC_ of absolute tap-to-tap' asynchrony A across trials in the CC was defined as follows:
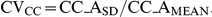
To make tap-to-tap' asynchrony comparable across CC trials with different regular self-paced tapping rates, the absolute asynchrony A_ii_ of each actual tap onset of T_i_ minus the expected tap onset T_ii_ was calculated as percent of the respective ITI_mean_:
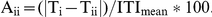
Note that this procedure is analogous to calculation of asynchrony A_i_ in the synchronization condition which takes into account the respective inter-onset-interval (IOI) of consecutive beats. Average asynchrony A in percent ITI_mean_ was calculated for each CC trial. Subsequently, CV_CC_ was determined across trials of each scan minutes as outlined above.

Analogous to the CV calculation in the SC task, the first two taps in each trial were excluded from further analysis.

As in the SC, only taps within the boundary of ±40% of the respective ITI_mean_ around the expected T_ii_ onset were considered.

Moreover, as in the SC, a repeated measures ANOVA with the factors SITE (PMv/AG), TMS (no TMS/TMS) and TIME (1^st^/4^th^ sub-block) was performed. In case significant interactions between SITE, TMS and TIME were present (p<0.05), post-hoc Bonferroni corrected t-tests were computed for CV_CC_ to test for differences between TMS and no TMS sub-blocks in PMv and AG sessions, respectively.

#### Tapping rate

Tapping rate was computed to control for differences in motor output between scans preceded or not preceded by rTMS. It was calculated by considering the overall number of taps per stimulus duration (6 sec) and was averaged across trials in each scan. A repeated measures ANOVA with the factors SITE (PMv/AG), TMS (TMS/no TMS) and CONDITION (SC/CC) was computed to probe the effect of TMS on overall tapping rate. To examine whether the subjects adjusted their tapping rate to the respective beat rate in the SC task independent of TMS sessions, a repeated measures ANOVA with the factors SITE (PMv/AG), TMS (TMS/no TMS) and BEAT RATE (1.7/2.0/2.5 Hz) was performed. In contrast to the above timing variability analysis, all taps produced during the stimulus presentation were included in the tapping rate analysis. Trials with tap corrections at the beginning of the trial, as well as trials during which subjects tapped twice as fast of the beat rate were not discarded from analysis of the tapping rate in order to control for potential changes in motor output and the overall tendency to couple motor responses to auditory input.

#### Error rate

SC and CC trials that contained at least one tap executed with an incorrect effector, i.e. a left finger tap when right finger tapping was cued at trial onset and vice versa, were used to compute the effector error rate as percent of all trials. A repeated measures ANOVA with the factors SITE (PMv/AG) and TMS (TMS/no TMS) was computed to examine the effect of TMS on error rate. As in the imaging data analysis, these trials were excluded from further statistics.

In case significance level of the Mauchly's test was below 0.05, Greenhouse-Geisser corrected values are reported.

### MRI data acquisition

Imaging was performed at a 3 T scanner (Siemens TRIO, Erlangen, Germany) equipped with a standard birdcage head coil. Participants were placed on the scanner bed in a supine position with their right and left index fingers positioned on a response button of the left and right response box. To prevent postural adjustments, the participants' arms and hands were carefully stabilized by supporting form-fitting cushions. Additional form-fitting cushions were utilized to prevent head and arm movements. Rhythms were presented over Nordic Neurolab AudioSystem headphones with 30 dB headset gradient noise attenuation. Further attenuation was achieved with insert earplugs rated to attenuate scanner noise by ∼38 dB. Thirty axial slices (210 mm field of view, 64×64 pixel matrix, 4 mm thickness; 1 mm spacing, in-plane resolution of 3.28×3.28 mm) positioned parallel to the bicomissural plane (AC-PC) covering the whole brain were acquired using a single-shot gradient echo-planar imaging (EPI) sequence (TE 30 ms, flip angle 90°, TR 2000 ms, 156.2 kHz acquisition bandwidth) sensitive to blood oxygenation level-dependent (BOLD) contrast. In total, 620 functional images were acquired in each single run. Prior to the functional imaging, 30 two-dimensional anatomical T1-weighted MDEFT images and 30 T1-weighted EPI images with the same spatial orientation as the functional data were acquired. The EPI acquisition was continuous to prevent periodic silent gaps between TRs to disrupt the participants' encoding of the rhythms. We chose a slice acquisition frequency of 15 Hz to ensure the continuous scanner noise to be well above the fastest frequency of elements of the rhythmical stimuli – beat subdivision – (12.5 Hz) to prevent an auditory interaction between the two sources of rhythmic patterns and ensure that the participants were able to attend to the stimuli. By conducting a short auditory test with the EPI sequence prior to data acquisition in each session we adjusted the sound level for each participant in such a way that the stimuli could be easily heard over the scanner noise by each participant at an individually comfortable sound pressure level. When explicitly asked in a post experimental interview, participants reported no difficulty hearing the stimuli during the whole course of the measurement or performing any of the tasks.

### MRI data analysis

Functional data were motion-corrected online with the Siemens motion correction protocol (Siemens, Erlangen, Germany). Further processing of the fMRI data was performed using the software package LIPSIA [52]. To correct for the temporal offset between the slices acquired in one image, a cubic-spline interpolation was employed. Low-frequency signal changes and baseline drifts were removed using a temporal highpass filter with a cutoff frequency of 1/96 Hz. Spatial smoothing was performed with a Gaussian filter of 5.65 mm FWHM. To align the functional data slices with a 3D stereotactic coordinate reference system, a rigid linear registration with six degrees of freedom (3 rotational, 3 translational) was performed. The rotational and translational parameters were acquired on the basis of the MDEFT [53] and EPI-T1 slices to achieve an optimal match between these slices and the individual 3D reference data set. This 3D reference data set was acquired for each subject during a previous scanning session. The MDEFT volume data set with 160 slices and 1 mm slice thickness was standardized to the Talairach stereotactic space [45]. The rotational and translational parameters were subsequently transformed by linear scaling to a standard size. The resulting parameters were then used to transform the functional slices using trilinear interpolation, so that the resulting functional slices were aligned with the stereotactic coordinate system, thus generating output data with a spatial resolution of 3×3×3 mm (27 mm^3^). The statistical evaluation was based on a least-squares estimation using the general linear model for serially autocorrelated observations [54]–[57]. The design matrix was generated with a synthetic hemodynamic response function [58], [59] and its first derivative modeled at the onset of the stimuli. Only trials in which all taps were performed with the correct effector according to cue (right or left hand, respectively) were included in the analysis. The number of taps during each stimulus was included as a regressor of no interest to control for differences between the number of taps in SC and CC. Resting trials were not included in the model. The model equation, including the observation data, the design matrix and the error term, was convolved with a Gaussian kernel of dispersion of 4 s FWHM to deal with the temporal autocorrelation [57]. In the following, contrast-images, i.e. beta value estimates of the raw-score differences between specified conditions, were generated for each participant. As noted before, each individual functional dataset was aligned with the standard stereotactic reference space, so that a group analysis based on the contrast-images could be performed. A one-sample t-test was employed for the group analyses across the contrast images of all subjects (SC vs. CC) for each of the four independent scans separately, which indicated whether observed differences between the two conditions were significantly distinct from zero. In addition, paired t-test of the same contrast images was performed to obtain statistical significance of pairwise comparisons between “PMv TMS” vs. “PMv no TMS”, “PMv TMS” vs. “AG TMS”, “PMv TMS” vs. “AG no TMS”. *T* values were subsequently transformed to *Z* scores. To compute the common activation increases in the above contrasts, a conjunction [60] between the contrasts SC vs. CC in all four scans, as well as between all pairwise comparisons was performed. Note that, since the current study is the first to evaluate short-term reorganization of the auditory motor integration network after rTMS, whole brain analysis were performed since our aim was to identify areas with a potential compensatory mechanism in addition to our hypothesis with regard to the role of the right PMv after rTMS of the left PMv.

To correct for false-positive results, in a first step, the initial voxelwise z-threshold was set to *Z* = 2.576 (p = .005, uncorrected) for the conjunction of the main contrast SC vs. CC across all scans, as well as *Z* = 2.33 (p = .01, uncorrected) for the conjunction of pairwise comparisons. In a second step, the results were corrected for multiple comparisons using cluster-size and cluster-value thresholds obtained by Monte-Carlo simulations at a significance level of p<.05. Based on our a priori hypothesis, activity increase in the right PMv after rTMS over the left PMv is reported on the basis of thresholded *Z* = 2.33 (p = .01), but uncorrected conjunction of the pairwise comparisons.

Additionally, we analyzed the signal change in several functionally defined regions of interest (ROIs). A ROI was defined as the peak voxel and a sphere of six adjacent voxels in regions of the dorsal auditory stream – the left PMv and the left STG that was activated relatively more for SC vs. CC, as well as in regions with significant activity increase after rTMS over the left PMv compared to all other scans – right PMv and cerebellar vermal lobule V. Within each ROI, the percentage signal change was calculated in relation to the mean signal intensity across all time steps of the respective scan and for each of the four five-minute sub-blocks to examine effects that change over time. Subsequently, the mean signal change over a 6 s epoch, starting 4 s after stimulus onset, was extracted for each condition and participant. To probe the compensatory nature of the activity enhancements after left PMv stimulation and determine the compensatory significance of these enhancements across time, multiple regression analyses were computed using the stepwise method for the respective ROIs, including individual percent signal increase for auditory-motor timing (SC minus CC) during each of the four five-minute sub-blocks after rTMS as potential predictors of the individual behavioral effect of rTMS (CV in TMS minus no TMS in the respective scan). Significant standardized regression coefficients are reported to assess the presence of an inverse relationship between the behavioral effect of rTMS and the activity increase for SC vs. CC after rTMS which would suggest a compensatory activity increase. Note that only predictors providing incremental explanation of behavioral variance (*P*-values≤0.05) entered the stepwise multiple regression model.

The anatomical locations of the functional activation were assigned by considering both the peak voxel and the position of the respective activation cluster on the mean brain of the 16 subjects transformed in Talairach stereotaxic space [45]. The MRI atlas of the cerebellum by Schmahmann et al. [61] was used to locate cerebellar activations.

## Results

### Behavioral results

#### Effect on auditory-motor timing: Coefficient of variation in the synchronization condition (CV_SC_)

**Figure 3:**
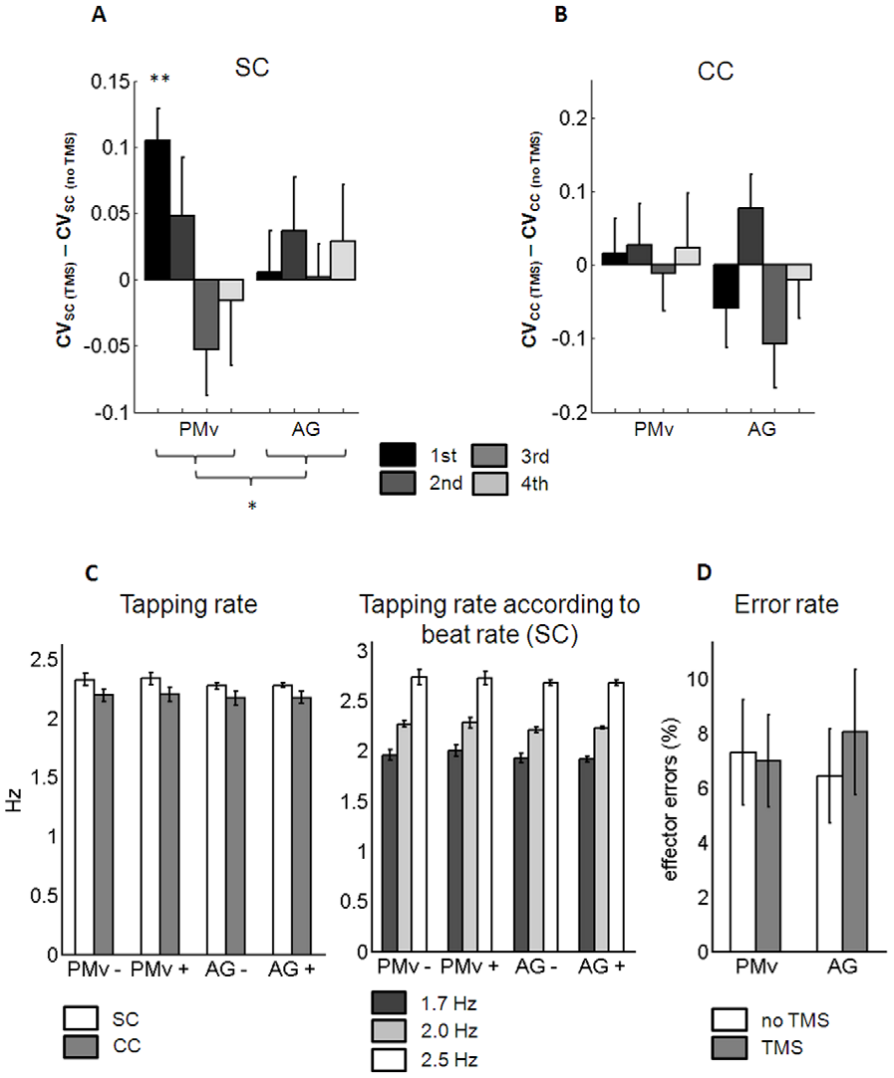
Behavioral effects of rTMS. **A**: Auditory-motor timing variability: Coefficient of variation of tap-to-beat asynchrony in the synchronization condition (CV_SC_) plotted as the difference between TMS and no TMS (baseline) for each of the four sub-blocks of the PMv and AG sessions, respectively. Results reveal a significant interaction between SITE (PMv/AG), TMS (no TMS/TMS) and TIME (1^st^/4^th^ sub-block) (* denotes p = 0.045 in a repeated measures ANOVA) due to an increase of CV_SC_ in the first sub-block after PMv stimulation (** denotes p<0.01, Bonferroni corrected). **B**: Control 1, Motor timing variability: Coefficient of variation of tap-to-tap' asynchrony in the self-paced control condition (CV_CC_) plotted as the difference between TMS and no TMS (baseline) for each of the four sub-blocks of the PMv and AG sessions, respectively. **C**: Control 2, Tapping rate in the SC and CC (left side) and tapping rate according to the beat rate of auditory rhythms in the SC (right side). “+” refers to the scan preceded by rTMS; “−” refers to the scan not preceded by rTMS. **D**: Control 3, Effector error rate. Error bars denote standard errors of the mean.

The coefficient of variation (CV) of tap-to-beat asynchrony across trials was taken as a measure of synchronization accuracy in accordance with Repp [36]. A repeated measures ANOVA revealed a significant interaction of SITE, TMS and TIME (F(1,15) = 4.79, p = .045) due to the difference between “PMv no TMS” and “PMv TMS” in the first sub-block of the PMv session (t(15) = −4.476, p<.01, Bonferroni corrected; Fig. 3A). The Kolmogorov-Smirnov tests confirmed that the assumption of normality was not violated (p>0.05).

#### Control 1 - Effect on motor timing: Coefficient of variability in the control condition (CV_CC_)

To ensure that this effect was related to auditory-motor timing and not just to motor timing variability, CV of tap-to-tap' asynchrony was computed in the control condition (CC), in which subject produced regular self-paced tapping during an auditory stimulus, that spectrally matched the auditory rhythms, where tap' is the time at which a tap should have occurred according to the mean inter-tap-interval in the respective trial (cf. Methods). In contrast to CV of tap-to-beat asynchrony in the SC condition, a repeated measures ANOVA with the factors SITE, TMS and TIME revealed no interaction between SITE, TMS and TIME (F(3,45) = 0.08, p = .781; Fig. 3B). The Kolmogorov-Smirnov tests confirmed that the assumption of normality was not violated (p>0.05).

#### Control 2 - Effect on motor output and on auditory-motor coupling: Tapping rate

Stimulation of the PMv might have potentially impaired primary motor function due to the possibility of stimulation spreading to primary motor cortex. To control for differences in motor output depending on the TMS session, the overall tapping rate was assessed for each session. A main effect of CONDITION (F(1,15) = 6.60, p = .021) due to slower overall tapping rate in the self-paced compared to the synchronization condition, but importantly, no interaction of SITE, TMS and CONDITION was found for tapping rate (F(1,15) = 0.01, p = .922; Fig. 3C, left side).

If rTMS had an influence on the overall coupling of the subjects' tapping rate to the auditory beat rate which could change from one SC trial to the next, they would be impaired in adjusting their tapping frequency to the beat rate of the respective rhythmic stimulus. However, a repeated measures revealed only a main effect of BEAT RATE (F(1.33,19.93) = 361.88, p<.01, Greenhouse-Geisser), but no interaction of SITE and TMS (F(1,15) = 0.14, p = .718), or SITE, TMS and BEAT RATE (F(2,30) = 1.35, p = .275; Fig. 3C, right side).

#### Control 3 - Effect on attention: Error rate

Any systematic effects of rTMS on attention in the SC condition could potentially influence the reported effect. The use of the non-cued hand, the effector error may indicate interference with motor plan selection and sound cue discrimination, or a decrease of attention to the task. However, a repeated measures ANOVA did not show any main effect or interaction between SITE and TMS with regard to error rate (Fig. 3D).

### fMRI results

#### Synchronization Condition (SC) vs. Control Condition (CC)

**Figure 4:**
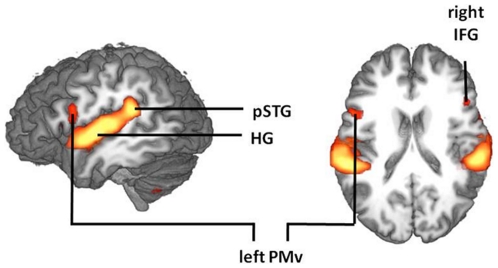
Synchronization (SC) versus self-paced control condition (CC). A conjunction of all four independent scans revealed activity increase in the bilateral Heschl's gyrus, the left posterior superior temporal gyrus and the left PMv for auditory-motor timing (SC vs. CC); corrected at p<.005, displayed at p<.001 for illustration purposes).

As expected, a conjunction of SC vs. CC in all four independent scans yielded an activity increase in the dorsal auditory stream comprising bilateral Heschl's gyrus (HG, BA 41/42), left posterior temporal gyrus (pSTG, BA 22) and left inferior ventral premotor cortex (PMv), as well as the posteriormost aspect of the pars opercularis (PMv, BA 6/44; Fig. 4). In addition, activity was enhanced in the right pars triangularis of the inferior frontal gyrus (IFG, BA 44/45), the right dorsal premotor cortex (PMd, BA 6), bilateral anterior insula (BA 13) and the cerebellar crus II (cf. Table 1 for Talairach coordinates).

**Table 1:** Anatomical specification, hemisphere (R right, L left), Talairach coordinates (x, y, z), volume (mm3) and maximal Z scores (*Z*) of significant activations in the direct contrasts.

Synchronization (SC) versus control condition (CC) in all four fMRI scans and activity increases after left PMv rTMS					
		Talairach coordinates			
Area	Brodmann area	*x*	*y*	*z*	*Z*
**SC>CC – Conjunction of four scans**					
L inferior ventral premotor cortex/precentral sulcus (PMv)	BA 6/44	−50	8	21	3.72
L Heschl's Gyrus (HG)	BA 41	−44	−13	3	6.52
	BA 41/42	−53	−22	12	6.41
R Heschl's Gyrus (HG)	BA 41	43	−10	3	7.32
	BA 42	61	−19	12	7.19
L posterior superior temporal gyrus (pSTG)/inferior parietal lobe (IPL)	BA 22/40	−56	−34	21	6.83
R dorsal premotor cortex (PMd)	BA6	46	2	48	4.26
R inferior frontal gyrus (IFG)	BA 44/45	46	17	12	3.58
L anterior insula	BA 13	−38	17	9	3.29
R anterior insula	BA 13	28	23	9	3.95
Cerebellum, Crus II	-	−26	−70	−36	3.73
**SC>CC – Conjunction of [“PMv TMS” vs. “PMv no TMS”], [“PMv TMS vs. AG TMS”] and [“PMv TMS vs. AG no TMS”]**					
R inferior ventral premotor cortex (PMv)	BA 6	58	2	18	3.21*
Cerebellum, vermal area V	-	1	−61	−6	3.29

#### Activation increases after PMv TMS during SC vs. CC

**Figure 5:**
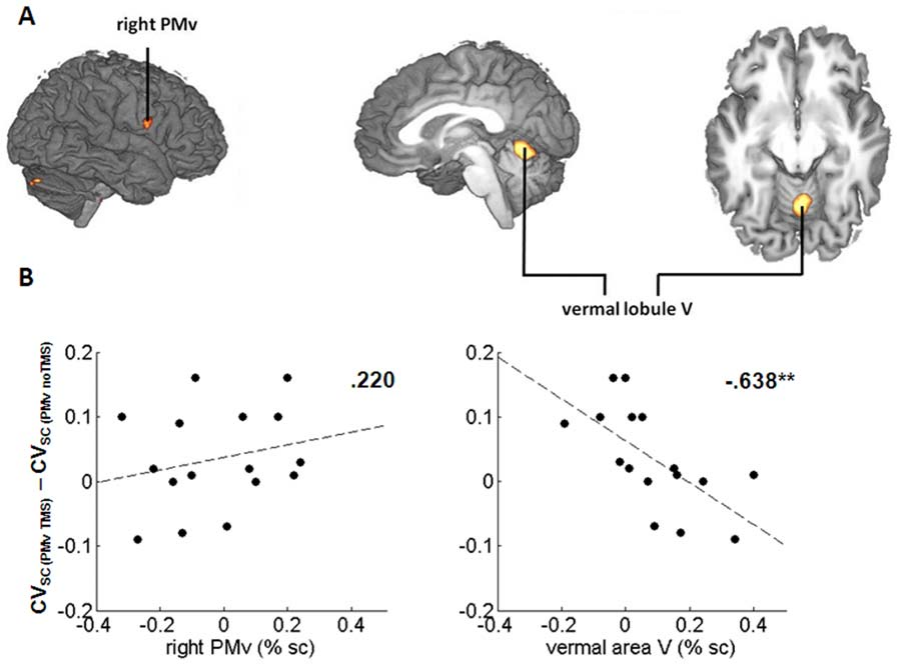
Compensatory activity following left PMv rTMS. **A:** rTMS over the left PMv triggered state-dependent activity increases (SC vs. CC, conjunction of rTMS PMv vs. no rTMS PMv, rTMS PMv vs. rTMS AG and rTMS PMv vs. no rTMS AG) in the right PMv (a priori hypothesis; uncorrected, displayed at p<.01) and the anterior midline cerebellum (corrected at p<.01). **B:** In contrast to right PMv activity (left side), the task-dependent cerebellar activity (right side) during the first five-minute sub-block following rTMS over the PMv predicted the preservation of auditory-motor synchronization accuracy (CV_SC_). Subjects with higher activity increase in the vermal lobule V following rTMS over the PMv were more likely to retain synchronization accuracy.

A conjunction of the contrasts between “PMv TMS” and all other scans for SC vs. CC allowed us to look for the effect of left PMv TMS on activity during auditory-motor integration. As hypothesized, rTMS over the left PMv stimulation was followed by a task-specific activation increase in the right inferior PMv. In addition, a significant activation increase was observed in the vermal area V of the anterior cerebellum (Fig. 5A).

The above stimulation-induced activity boosts in the right PMv and the anterior cerebellum revealed a differential role of these two areas in the preservation of synchronization accuracy. Linear stepwise multiple regression analyses with percent signal change for SC vs. CC during five-minute sub-blocks (1^st^, 2^nd^, 3^rd^, 4^th^) as predictors for the behavioral effect of left PMv stimulation were computed to test whether these changes are compensatory or unspecific effects of rTMS over the left PMv. None of the four sub-blocks of right PMv activity significantly predicted the subjects' behavioral performance. In contrast, the vermal area of the anterior cerebellum explained 40% variance of the behavioral effect of rTMS over the left PMv (multiple regression coefficient *R* = .638, p<.001): the higher the percent signal change in the first five minutes following TMS, the smaller was the effect of left PMv stimulation on synchronization accuracy (standardized regression coefficient *beta* = −.638; Fig. 5B). Thus, task-specific cerebellar activity in the first five minutes after rTMS predicted how much the subject's synchronization accuracy would be preserved in the “PMv TMS” scan, indicating a compensatory mechanism of this region in auditory-motor synchronization accuracy.

Notably, neither the right PMv nor the vermal area of the cerebellum predicted the behavioral changes of AG TMS compared to baseline.

## Discussion

The present combined rTMS-fMRI experiment probed the critical role of the left ventral premotor cortex (PMv) in auditory-motor timing, and investigated task-dependent activity increases that help to preserve auditory-motor synchronization following its disruption. Subjects synchronized left or right finger tapping to the beat rates (1.7, 2.0, 2.5 Hz) of auditory rhythms (synchronization condition; SC), and produced regular self-paced tapping during spectrally identical, but temporally scrambled versions of the same rhythms (control condition; CC). Results demonstrated that rTMS over the PMv, but not over a control region, temporarily disrupted auditory-motor timing, leaving motor timing variability, primary motor function and attention to task and stimuli intact. Moreover, it triggered task-dependent activity increases in the right PMv contralateral to the stimulation and the anterior midline cerebellum. In contrast to right PMv activity, cerebellar activity at the beginning of the scan predicted how much auditory-motor synchronization accuracy would be affected, with higher activity in less impaired subjects.

In the synchronization condition (SC), the beat rate of the auditory rhythms could change on a trial-to-trial basis. Accordingly, the task required the subjects to predict the onset of the upcoming isochronous beats on the basis of the first two beats of the respective rhythmic stimulus and align their finger tapping to the predicted onsets accordingly. Accurate auditory-motor timing requires resources that enable precise feed-forward prediction of ex-afferent auditory and re-afferent somatosensory and proprioceptive feedback. This feed-forward interpretation is in line with the assumption that the perception of beats and the generation of taps rely on a shared central timeline [62]. Importantly, while tap-to-beat asynchrony was affected during the first sub-block after stimulation, self-paced tapping remained intact. This suggests that specifically the variability of *auditory-motor timing*, but not that of self-paced motor timing was impaired after rTMS over the PMv. Moreover, these results could not be explained by a degradation of motor output, or overall auditory-motor coupling, since the subjects adjusted their tapping rate to the respective beat rate independent of the rTMS condition. Finally, rTMS had no effect on the effector error rate, which renders an impairment of the subjects' attention to the task, motor plan selection or sound cue discrimination unlikely.

The present behavioral results suggest that the left inferior PMv is part of a network critically involved in auditory-motor timing. This result is in line with the present fMRI findings (cf. Fig. 4), as well as with previous imaging studies reporting this region within the context of auditory-motor synchronization (e.g. Rao et al., 1997; Jancke et al., 2000; Thaut, 2003). However, other regions were also shown to be critically involved in right hand [25], [26], [28] as well as both right and left hand [29] tapping synchronization such as the lateral cerebellum and left dorsal premotor cortex. The present findings may substantiate an effector-independent impairment of auditory-motor timing, since subjects performed tapping to an auditory beat with their left or right hand on a trial-by-trial basis. Yet, direct evidence for this assumption may be provided only with a study that allows to test effects of PMv disruption on each effector, separately. An effect of PMv disruption on effector-independent auditory-motor timing is consistent with results showing that some premotor grasping neurons are unspecific for limb and grip type, i.e. grasping with left or right hand or the mouth can engage the same neurons [64] and back up the framework proposing that rhythmic prediction is sub-served by an audiomotor fraction of a full-blown action representation [11].

Ruspantini et al. [63] provided recent evidence for a critical involvement of the PMv in visuo-motor synchronization. As in the current study, the effect of PMv disruption was specific to externally paced in contrast to self-paced timing. Together, the latter and the current studies suggest that the PMv is a critical node for modality-independent externally paced motor timing.

Although externally- and self-paced timing show considerable overlap of underlying neural networks [19], [65], PMv stimulation disrupted only auditorily paced motor timing, whereas internally paced motor timing was spared. This dissociation supports the notion of a lateral premotor cortex dominance over externally guided movements and a medial premotor cortex dominance over internally guided movements [66], [67]. The critical role of the PMv in auditory-motor timing suggested in the current study may explain the therapeutic effects of external beat stimulation on gait and speech reported in PD patients [68]–[73]. External pacing may less strongly recruit the disrupted medial premotor-basal ganglia loop as opposed to the intact PMv which consequently helps to alleviate deficits in motor timing.

Note that Malcolm and colleagues (2008) did not find a significant effect of rTMS over the left PMv on auditory-motor synchronization. This might be partly explained by different measures of synchronization, such as absolute tap-to-beat asynchrony in the above study [74] and synchronization accuracy in the present study. Moreover, in contrast to the former, the beats used here were surrounded by other rhythmic events (Fig. 1), as it is the case in music. Compared to metronome clicks, they require more attentional resources to be directed to the auditory modality.

The variability of tap-to-beat synchronization was impaired after repetitive TMS interference with a region of the left dorsal auditory stream, which is frequently regarded as tuned to language functions. In all four independent fMRI-scans, the SC versus the CC yielded activity in regions of the dorsal-auditory stream – the bilateral Heschl's gyrus, the posterior superior temporal gyrus (pSTG) and the PMv. Remarkably, the pSTG and the PMv were left-lateralized. Comparable activity increases are considered to be related to speech perception and production [1], [3]. However, like speech, a non-speech task such as synchronization of finger tapping to an auditory beat requires a temporally precise prediction of auditory, somatosensory and motor information. The enhanced recruitment of bilateral primary auditory, left temporo-parietal and premotor regions reveals that synchronization of left and right finger tapping to a musical beat exploits a circuit that is otherwise involved in vocalization and speech. Note that this pattern of activity is unlikely to be caused by increased sub-vocalization during the synchronization condition. There was no consistent activity increase in areas characteristic for motor imagery and motor preparation such as the primary motor cortex, primary and secondary somatosensory areas, and in particular the SMA/pre-SMA, an area which has been shown to be most reliably involved in vocal imagery [23], [24], [75], [76].

As hypothesized, activity in the homologue right PMv (contralateral to the stimulated site) was enhanced for auditory-motor timing following rTMS over the left PMv in comparison to all other scans. This result is in line with evidence provided by both stroke and rTMS studies, which demonstrated task-dependent activity increase in non-dominant homologue regions contralateral to the affected primary motor, premotor or prefrontal sites during motor, visuo-motor and speech tasks [31], [32], [35]. Such activity boosts are hypothesized to arise from decreased transcallosal inhibition that occurs as a result of the disruption of the respective area in the left or the right hemispheres: Although callosal fibers are predominantly excitatory [77], [78], transcallosal inhibition is thought to be mediated by these excitatory fibers projecting onto GABA-ergic inhibitory neurons [79].

Despite the occurrence of the hypothesized task-specific increase in the right PMv after left PMv stimulation, our assumption concerning the compensatory nature of this activity was not supported. None of the four five-minute sub-blocks of the scan following left PMv stimulation significantly explained the effect of the latter on the subjects' auditory-motor synchronization. This result lends support to studies demonstrating no compensatory or even adverse behavioral effects of activity increase in the non-dominant hemisphere contralateral to the affected region [32]–[35]. The functional relevance of the enhanced recruitment of contralesional primary motor and premotor cortex is, however, still under debate [35], [80], [81].

Besides the right PMv, an extended region in the midline anterior cerebellar lobe (vermal lobule V) was more strongly activated during SC when preceded by rTMS over the left PMv compared to all other scans. Importantly, in contrast to the right PMv, activity in the initial five minutes after rTMS in the anterior cerebellar lobe reliably predicted how well subjects preserved auditory-motor synchronization accuracy during the scan following left PMv stimulation. This covariance was specific to TMS over the left PMv and not present following TMS over the left AG (control site).

Unlike the right PMv which is interconnected with the left PMv via transcallosal fibers, the vermal lobule V is not known to have pronounced multi-synaptic projections to the left PMv. Kelly and Strick [82] induced retrograde tracers into the adjacent frontal motor site – arm area of the primary motor cortex – and found only few labeled Purkinje cells in the vermal lobules IV–VI, with most clusters beginning 4 mm from the midline. Moreover, in contrast to the cerebellar hemispheres, which project to the dentate nucleus that shows distinct output to the PMv [83], the vermal lobule projects to the fastigial nucleus [84].

Notably, the output of the midline cerebellum, the fastigial nucleus, has been proposed to serve as an interface between cerebro-cerebellar and spino-cerebellar loops, i.e. as a comparator between top-down motor commands and bottom-up visual, vestibular, proprioceptive and exteroceptive feedback signals which provide information on the current state of the system [85]. Support for the role of the anterior vermal lobe in the temporal integration of multimodal information is provided by neuroimaging: While generally sensorimotor in contrast to cognitive tasks are known to activate the anterior part of the cerebellum [86], the anterior vermal region has been proposed in temporal processing of multisensory, e.g. tactile and proprioceptive, information [87]. Consistent with the current motor timing task, Spencer and colleagues [88] demonstrated a recruitment of this area in discrete in contrast to continuous timing, which contains a pause inserted before each flexion phase, such as rhythmic finger tapping in the current experiment. In line with the current data revealing an inverse relationship of anterior vermal lobe activity and the impairment of tap-to-beat asynchrony, this area has been recently associated with reduced reaction time (RT) variability (RT coefficient of variation) in children [89]. Finally, this region has also been reported during reduced predictability of visual [90] and somatosensory [91] sequences, increased difficulty of temporal auditory tasks, as well as perception and production of complex versus isochronous visual rhythms [92].

Taken together, the short-term task-dependent compensatory activation of the vermal area V suggests that more resources were devoted to temporal mismatch detection between bottom-up (auditory and somatosensory input) and top-down (corollary discharges of motor output) information after interference with the left PMv. This activity increase occurred only following the disruption of the PMv, which is a causal node in auditory-motor information timing, but not the control region, which underlines the causal involvement of the left PMv in auditory-motor timing. In subjects with a more pronounced activity increase in the vermal area V following PMv stimulation, the deteriorating effect of PMv stimulation on synchronization accuracy was mitigated.

It cannot be deduced from the present findings whether the enhanced temporal mismatch detection between these information channels may have occurred due to altered (i) top-down, via remote influence of rTMS over the left PMv, (ii) bottom-up, via the behavioral impairment following left PMv stimulation or (iii) interaction of top-down and bottom-up information. Furthermore, to probe whether the compensatory metabolic activity in the anterior midline cerebellum reflects a more general, supramodal mechanism of temporal mismatch detection, future studies should test whether it is bound to auditory cues or occurs during synchronization to visual and somatosensory cues, as well.

In conclusion, the left PMv critically contributes to auditory-motor timing. Repetitive TMS interference with its activity triggers differential compensatory mechanisms in remote sites: While task-specific activity increase in the right PMv contralateral to the stimulated region does not help to retain behavior, activity in the anterior cerebellum can be linked to a transiently effective compensation of auditory-motor timing after PMv disruption.

## Supporting Information

Sounds S1 Six examples of the 216 musical rhythms. The filenames denote the properties of the rhythms as follows: e.g. “34a100_D.wav” refers to a musical rhythm with three beats per measure, four elements per beat, a long-short rhythmic figure, a slow tempo (100 BPM) consisting of predominantly metal drum sounds (“metallic”). A and B are the two instrumental versions of “wooden”, C and D the two instrumental versions of “metallic”.
(MP3)

Sounds S2 Six examples of the 216 musical rhythms. The filenames denote the properties of the rhythms as follows: e.g. “34a100_D.wav” refers to a musical rhythm with three beats per measure, four elements per beat, a long-short rhythmic figure, a slow tempo (100 BPM) consisting of predominantly metal drum sounds (“metallic”). A and B are the two instrumental versions of “wooden”, C and D the two instrumental versions of “metallic”.
(MP3)

Sounds S3 Six examples of the 216 musical rhythms. The filenames denote the properties of the rhythms as follows: e.g. “34a100_D.wav” refers to a musical rhythm with three beats per measure, four elements per beat, a long-short rhythmic figure, a slow tempo (100 BPM) consisting of predominantly metal drum sounds (“metallic”). A and B are the two instrumental versions of “wooden”, C and D the two instrumental versions of “metallic”.
(MP3)

Sounds S4 Six examples of the 216 musical rhythms. The filenames denote the properties of the rhythms as follows: e.g. “34a100_D.wav” refers to a musical rhythm with three beats per measure, four elements per beat, a long-short rhythmic figure, a slow tempo (100 BPM) consisting of predominantly metal drum sounds (“metallic”). A and B are the two instrumental versions of “wooden”, C and D the two instrumental versions of “metallic”.
(MP3)

Sounds S5 Six examples of the 216 musical rhythms. The filenames denote the properties of the rhythms as follows: e.g. “34a100_D.wav” refers to a musical rhythm with three beats per measure, four elements per beat, a long-short rhythmic figure, a slow tempo (100 BPM) consisting of predominantly metal drum sounds (“metallic”). A and B are the two instrumental versions of “wooden”, C and D the two instrumental versions of “metallic”.
(MP3)

Sounds S6 Six examples of the 216 musical rhythms. The filenames denote the properties of the rhythms as follows: e.g. “34a100_D.wav” refers to a musical rhythm with three beats per measure, four elements per beat, a long-short rhythmic figure, a slow tempo (100 BPM) consisting of predominantly metal drum sounds (“metallic”). A and B are the two instrumental versions of “wooden”, C and D the two instrumental versions of “metallic”.
(MP3)

Sound S7 Examples of several experimental and control trials.
(MP3)

## References

[1] Hickok G, Poeppel D (2007). The cortical organization of speech processing.. Nat Rev Neurosci.

[2] Zatorre RJ, Chen JL, Penhune VB (2007). When the brain plays music: auditory-motor interactions in music perception and production.. Nat Rev Neurosci.

[3] Rauschecker JP, Scott SK (2009). Maps and streams in the auditory cortex: nonhuman primates illuminate human speech processing.. Nat Neurosci.

[4] Catani M, Mesulam M (2008). The arcuate fasciculus and the disconnection theme in language and aphasia: history and current state.. Cortex.

[5] Schubotz RI, Anwander A, Knoesche TR, von Cramon DY, Tittgemeyer M (2010). Anatomical and functional parcellation of the human lateral premotor cortex.. Neuroimage.

[6] Rilling JK, Glasser MF, Preuss TM, Ma X, Zhao T (2008). The evolution of the arcuate fasciculus revealed with comparative DTI.. Nat Neurosci.

[7] Bernal B, Ardila A (2009). The role of the arcuate fasciculus in conduction aphasia.. Brain.

[8] Dum RP, Strick PL (1991). The origin of corticospinal projections from the premotor areas in the frontal lobe.. J Neurosci.

[9] Dum RP, Strick PL (2002). Motor areas in the frontal lobe of the primate.. Physiol Behav.

[10] Dancause N, Barbay S, Frost SB, Mahnken JD, Nudo RJ (2007). Interhemispheric connections of the ventral premotor cortex in a new world primate.. J Comp Neurol.

[11] Schubotz RI (2007). Prediction of external events with our motor system: towards a new framework.. Trends Cogn Sci.

[12] Coull J, Vidal F, Goulon C, Nazarian B, Craig C (2008). Using Time-to-Contact Information to Assess Potential Collision Modulates Both Visual and Temporal Prediction Networks.. Front Hum Neurosci.

[13] O'Reilly JX, Mesulam MM, Nobre AC (2008). The cerebellum predicts the timing of perceptual events.. J Neurosci.

[14] Schubotz RI, Friederici AD, von Cramon DY (2000). Time perception and motor timing: a common cortical and subcortical basis revealed by fMRI.. Neuroimage.

[15] Schubotz RI, von Cramon DY (2001). Interval and ordinal properties of sequences are associated with distinct premotor areas.. Cereb Cortex.

[16] Schubotz RI, von Cramon DY, Lohmann G (2003). Auditory what, where, and when: a sensory somatotopy in lateral premotor cortex.. Neuroimage.

[17] Wolfensteller U, Schubotz RI, von Cramon DY (2007). Understanding non-biological dynamics with your own premotor system.. Neuroimage.

[18] Chen JL, Penhune VB, Zatorre RJ (2008). Listening to Musical Rhythms Recruits Motor Regions of the Brain.. Cereb Cortex.

[19] Rao SM, Harrington DL, Haaland KY, Bobholz JA, Cox RW (1997). Distributed neural systems underlying the timing of movements.. J Neurosci.

[20] Jancke L, Loose R, Lutz K, Specht K, Shah NJ (2000). Cortical activations during paced finger-tapping applying visual and auditory pacing stimuli.. Brain Res Cogn Brain Res.

[21] Thaut MH (2003). Neural basis of rhythmic timing networks in the human brain.. Ann N Y Acad Sci.

[22] Chen JL, Penhune VB, Zatorre RJ (2009). The role of auditory and premotor cortex in sensorimotor transformations.. Ann N Y Acad Sci.

[23] Riecker A, Ackermann H, Wildgruber D, Dogil G, Grodd W (2000). Opposite hemispheric lateralization effects during speaking and singing at motor cortex, insula and cerebellum.. Neuroreport.

[24] Kleber B, Birbaumer N, Veit R, Trevorrow T, Lotze M (2007). Overt and imagined singing of an Italian aria.. Neuroimage.

[25] Théoret H (2001). Increased variability of paced finger tapping accuracy following repetitive magnetic stimulation of the cerebellum in humans.. Neuroscience Letters.

[26] Doumas M, Praamstra P, Wing AM (2005). Low frequency rTMS effects on sensorimotor synchronization.. Exp Brain Res.

[27] Chen JL, Zatorre RJ, Penhune VB (2006). Interactions between auditory and dorsal premotor cortex during synchronization to musical rhythms.. Neuroimage.

[28] Del Olmo MF, Cheeran B, Koch G, Rothwell JC (2007). Role of the cerebellum in externally paced rhythmic finger movements.. J Neurophysiol.

[29] Pollok B, Rothkegel H, Schnitzler A, Paulus W, Lang N (2008). The effect of rTMS over left and right dorsolateral premotor cortex on movement timing of either hand.. Eur J Neurosci.

[30] Chollet F, DiPiero V, Wise RJ, Brooks DJ, Dolan RJ (1991). The functional anatomy of motor recovery after stroke in humans: a study with positron emission tomography.. Ann Neurol.

[31] O'Shea J, Johansen-Berg H, Trief D, Gobel S, Rushworth MF (2007). Functionally specific reorganization in human premotor cortex.. Neuron.

[32] Kell CA, Neumann K, von Kriegstein K, Posenenske C, von Gudenberg AW (2009). How the brain repairs stuttering.. Brain.

[33] Liepert J, Zittel S, Weiller C (2007). Improvement of dexterity by single session low-frequency repetitive transcranial magnetic stimulation over the contralesional motor cortex in acute stroke: a double-blind placebo-controlled crossover trial.. Restor Neurol Neurosci.

[34] Grefkes C, Nowak DA, Eickhoff SB, Dafotakis M, Kust J (2008). Cortical connectivity after subcortical stroke assessed with functional magnetic resonance imaging.. Ann Neurol.

[35] Nowak DA, Grefkes C, Dafotakis M, Eickhoff S, Küst J (2008). Effects of low-frequency repetitive transcranial magnetic stimulation of the contralesional primary motor cortex on movement kinematics and neural activity in subcortical stroke.. Arch Neurol.

[36] Repp BH (2005). Sensorimotor synchronization: a review of the tapping literature.. Psychon Bull Rev.

[37] Oldfield RC (1971). The assessment and analysis of handedness: the Edinburgh inventory.. Neuropsychologia.

[38] Peretz I, Champod AS, Hyde K (2003). Varieties of musical disorders. The Montreal Battery of Evaluation of Amusia.. Ann N Y Acad Sci.

[39] Kornysheva K, von Anshelm-Schiffer A-M, Schubotz RI (2010). Inhibitory stimulation of the ventral premotor cortex temporarily interferes with musical beat rate preference.. Hum Brain Mapp.

[40] Wassermann EM (1998). Risk and safety of repetitive transcranial magnetic stimulation: report and suggested guidelines from the International Workshop on the Safety of Repetitive Transcranial Magnetic Stimulation, June 5–7, 1996.. Electroencephalogr Clin Neurophysiol.

[41] Rossi S, Hallett M, Rossini PM, Pascual-Leone A, of TMS Consensus Group S (2009). Safety, ethical considerations, and application guidelines for the use of transcranial magnetic stimulation in clinical practice and research.. Clin Neurophysiol.

[42] Kornysheva K, von Cramon DY, Jacobsen T, Schubotz RI (2010). Tuning-in to the beat: Aesthetic appreciation of musical rhythms correlates with a premotor activity boost.. Hum Brain Mapp.

[43] Fitzgerald PB, Fountain S, Daskalakis ZJ (2006). A comprehensive review of the effects of rTMS on motor cortical excitability and inhibition.. Clin Neurophysiol.

[44] Allen EA, Pasley BN, Duong T, Freeman RD (2007). Transcranial magnetic stimulation elicits coupled neural and hemodynamic consequences.. Science.

[45] Talairach J, Tournoux P (1988). Co-Planar Stereotaxic Atlas of the Human Brain.

[46] Awiszus F (2003). TMS and threshold hunting.. Suppl Clin Neurophysiol.

[47] Awiszus F, Feistner H, Urbach D, Bostock H (1999). Characterisation of paired-pulse transcranial magnetic stimulation conditions yielding intracortical inhibition or I-wave facilitation using a threshold-hunting paradigm.. Exp Brain Res.

[48] Mishory A, Molnar C, Koola J, Li X, Kozel FA (2004). The maximum-likelihood strategy for determining transcranial magnetic stimulation motor threshold, using parameter estimation by sequential testing is faster than conventional methods with similar precision.. J ECT.

[49] Rossini PM, Barker AT, Berardelli A, Caramia MD, Caruso G (1994). Non-invasive electrical and magnetic stimulation of the brain, spinal cord and roots: basic principles and procedures for routine clinical application. Report of an IFCN committee.. Electroencephalogr Clin Neurophysiol.

[50] Rothwell JC, Hallett M, Berardelli A, Eisen A, Rossini P (1999). Magnetic stimulation: motor evoked potentials. The International Federation of Clinical Neurophysiology.. Electroencephalogr Clin Neurophysiol Suppl.

[51] Mills KR, Nithi KA (1997). Corticomotor threshold to magnetic stimulation: normal values and repeatability.. Muscle Nerve.

[52] Lohmann G, Muller K, Bosch V, Mentzel H, Hessler S (2001). LIPSIA - a new software system for the evaluation of functional magnetic resonance images of the human brain.. Comput Med Imaging Graph.

[53] Norris DG (2000). Reduced power multislice MDEFT imaging.. J Magn Reson Imaging.

[54] Friston KJ (1994). Statistical parametric mapping.. Functional neuroimaging: technical foundations.

[55] Friston KJ, Holmes AP, Poline JB, Grasby PJ, Williams SC (1995). Analysis of fMRI time-series revisited.. Neuroimage.

[56] Friston KJ, Holmes AP, Worsley KJ, Poline JB, Frith CD (1995). Statistical parametric maps in functional imaging: a general linear approach.. Hum Brain Mapp.

[57] Worsley KJ, Friston KJ (1995). Analysis of fMRI time-series revisited-again.. Neuroimage.

[58] Josephs O, Turner R, Friston KJ (1997). Event-related fMRI.. Hum Brain Mapp.

[59] Friston KJ, Fletcher P, Josephs O, Holmes A, Rugg MD (1998). Event-related fMRI: characterizing differential responses.. Neuroimage.

[60] Nichols T, Brett M, Andersson J, Wager T, Poline J-B (2005). Valid conjunction inference with the minimum statistic.. Neuroimage.

[61] Schmahmann JD, Doyon J, Toga AW, Petrides M, Evans AC (2000). MRI Atlas of the Human Cerebellum.

[62] Aschersleben G, Prinz W (1995). Synchronizing actions with events: the role of sensory information.. Percept Psychophys.

[63] Ruspantini I, Mäki H, Korhonen R, D'Ausilio A, Ilmoniemi RJ (2011). The functional role of the ventral premotor cortex in a visually paced finger tapping task: a TMS study.. Behav Brain Res.

[64] Rizzolatti G, Gentilucci M, Fogassi L, Luppino G, Matelli M (1987). Neurons related to goal-directed motor acts in inferior area 6 of the macaque monkey.. Exp Brain Res.

[65] Witt ST, Laird AR, Meyerand ME (2008). Functional neuroimaging correlates of finger-tapping task variations: An ALE meta-analysis.. NeuroImage.

[66] Goldberg G (1985). Supplementary motor area structure and function: Review and hypotheses.. Behav Brain Sci.

[67] Schubotz RI, von Cramon DY (2003). Functional-anatomical concepts of human premotor cortex: evidence from fMRI and PET studies.. Neuroimage.

[68] McIntosh GC, Brown SH, Rice RR, Thaut MH (1997). Rhythmic auditory-motor facilitation of gait patterns in patients with Parkinson's disease.. J Neurol Neurosurg Psychiatry.

[69] Thaut MH, Kenyon GP, Schauer ML, McIntosh GC (1999). The connection between rhythmicity and brain function.. IEEE Eng Med Biol Mag.

[70] Thaut MH, McIntosh KW, McIntosh GC, Hoemberg V (2001). Auditory rhythmicity enhances movement and speech motor control in patients with Parkinson's disease.. Funct Neurol.

[71] van Wegen E, Lim I, de Goede C, Nieuwboer A, Willems A (2006). The effects of visual rhythms and optic flow on stride patterns of patients with Parkinson's disease.. Parkinsonism Relat Disord.

[72] Willems AM, Nieuwboer A, Chavret F, Desloovere K, Dom R (2007). Turning in Parkinson's disease patients and controls: the effect of auditory cues.. Mov Disord.

[73] Baker K, Rochester L, Nieuwboer A (2008). The effect of cues on gait variability - reducing the attentional cost of walking in people with Parkinson's disease.. Parkinsonism Relat Disord.

[74] Malcolm MP, Lavine A, Kenyon G, Massie C, Thaut M (2008). Repetitive transcranial magnetic stimulation interrupts phase synchronization during rhythmic motor entrainment.. Neurosci Lett.

[75] Kawashima R, Okuda J, Umetsu A, Sugiura M, Inoue K (2000). Human cerebellum plays an important role in memory-timed finger movement: an fMRI study.. J Neurophysiol.

[76] Thobois S, Dominey PF, Decety J, Pollak PP, Gregoire MC (2000). Motor imagery in normal subjects and in asymmetrical Parkinson's disease: a PET study.. Neurology.

[77] Innocenti GM (1986). The general organization of callosal connections inthe cerebral cortex.. Cerebral Cortex.

[78] Bloom JS, Hynd GW (2005). The role of the corpus callosum in interhemispheric transfer of information: excitation or inhibition?. Neuropsychol Rev.

[79] Ferbert A, Priori A, Rothwell JC, Day BL, Colebatch JG (1992). Interhemispheric inhibition of the human motor cortex.. J Physiol.

[80] Murase N, Duque J, Mazzocchio R, Cohen LG (2004). Influence of interhemispheric interactions on motor function in chronic stroke.. Ann Neurol.

[81] Stinear CM, Barber PA, Smale PR, Coxon JP, Fleming MK (2007). Functional potential in chronic stroke patients depends on corticospinal tract integrity.. Brain.

[82] Kelly RM, Strick PL (2003). Cerebellar loops with motor cortex and prefrontal cortex of a nonhuman primate.. J Neurosci.

[83] Strick PL, Dum RP, Fiez JA (2009). Cerebellum and nonmotor function.. Annu Rev Neurosci.

[84] Habas C, Kamdar N, Nguyen D, Prater K, Beckmann CF (2009). Distinct cerebellar contributions to intrinsic connectivity networks.. J Neurosci.

[85] Mori S, Nakajima K, Mori F, Matsuyama K (2004). Integration of multiple motor segments for the elaboration of locomotion: role of the fastigial nucleus of the cerebellum.. Prog Brain Res.

[86] Stoodley CJ, Schmahmann JD (2009). Functional topography in the human cerebellum: a meta-analysis of neuroimaging studies.. Neuroimage.

[87] Kavounoudias A, Roll JP, Anton JL, Nazarian B, Roth M (2008). Proprio-tactile integration for kinesthetic perception: an fMRI study.. Neuropsychologia.

[88] Spencer RMC, Verstynen T, Brett M, Ivry R (2007). Cerebellar activation during discrete and not continuous timed movements: an fMRI study.. Neuroimage.

[89] Simmonds DJ, Fotedar SG, Suskauer SJ, Pekar JJ, Denckla MB (2007). Functional brain correlates of response time variability in children.. Neuropsychologia.

[90] Toma K, Ozawa M, Matsuo K, Nakai T, Fukuyama H (2003). The role of the human supplementary motor area in reactive motor operation.. Neurosci Lett.

[91] Tesche CD, Karhu JJ (2000). Anticipatory cerebellar responses during somatosensory omission in man.. Hum Brain Mapp.

[92] Xu D, Liu T, Ashe J, Bushara KO (2006). Role of the olivo-cerebellar system in timing.. J Neurosci.

